# Advancing Diagnosis and Treatment in People Living with HIV and Tuberculosis Meningitis

**DOI:** 10.1007/s11904-023-00678-6

**Published:** 2023-11-10

**Authors:** Sarah Kimuda, Derrick Kasozi, Suzan Namombwe, Jane Gakuru, Timothy Mugabi, Enock Kagimu, Morris K. Rutakingirwa, Kristoffer E. Leon, Felicia Chow, Sean Wasserman, David R. Boulware, Fiona V. Cresswell, Nathan C. Bahr

**Affiliations:** 1 Infectious Diseases Institute, Makerere University, Kampala, Uganda; 2 Departments of Neurology and Medicine (Infectious Diseases), University of California San Francisco, San Francisco, CA USA; 3 Institute for Infection and Immunity, St George’s, University of London, London, United Kingdom; 4 Wellcome Centre for Infectious Diseases Research in Africa, Institute of Infectious Disease and Molecular Medicine, University of Cape Town, Cape Town, South Africa; 5 Division of Infectious Diseases and International Medicine, Department of Medicine, University of Minnesota, Minneapolis, MN USA; 6 HIV Interventions, MRC/UVRI-LSHTM Uganda Research Unit, Entebbe, Uganda; 7 Global Health and Infection, Brighton and Sussex Medical School, Brighton, UK; 8 Division of Infectious Diseases, Department of Medicine, University of Kansas Medical Center, Kansas City, KS USA

**Keywords:** Tuberculous meningitis, Tuberculosis, TB, Diagnostic tests, Central nervous system infection

## Abstract

**Purpose of review:**

Tuberculous meningitis (TBM) is the most severe form of tuberculosis. Inadequate diagnostic testing and treatment regimens adapted from pulmonary tuberculosis without consideration of the unique nature of TBM are among the potential drivers. This review focuses on the progress being made in relation to both diagnosis and treatment of TBM, emphasizing promising future directions.

**Recent findings:**

The molecular assay GeneXpert MTB/Rif Ultra has improved sensitivity but has inadequate negative predictive value to “rule-out” TBM. Evaluations of tests focused on the host response and bacterial components are ongoing. Clinical trials are in progress to explore the roles of rifampin, fluoroquinolones, linezolid, and adjunctive aspirin.

**Summary:**

Though diagnosis has improved, novel modalities are being explored to improve the rapid diagnosis of TBM. Multiple ongoing clinical trials may change current therapies for TBM in the near future.

## Introduction

Tuberculosis (TB) meningitis is the most severe form of TB with an estimated 164,000 cases among adults in 2019 [1••]. In an estimate including undiagnosed cases, mortality was up to 70% among people living with HIV (PLWH) and up to 40% in people without HIV [1••]. If only cases that actually received treatment were included, mortality was still 60% in PLWH and 18% in people without HIV [1••]. Further, among survivors, neurological disability is common, estimated as up to ~30% in one systematic review [2•].

Two major contributors to poor outcomes in TBM are 1) diagnosis remains difficult and diagnostic tests imperfect and 2) even if the diagnosis is made rapidly and accurately, TBM treatment regimens have largely been adapted (with little change) from pulmonary TB regimens. It is unclear if regimens designed for pulmonary TB are actually the best regimens to treat TB meningitis. This review will consider both issues in depth summarizing the current state of affairs, recent innovations, and future directions that may lead to improved outcomes.

## TBM Prevention

Ideally, given the poor outcomes, TBM would be prevented. In order to prevent TBM once an individual is infected with TB, recognition of TB prior to its spread to the central nervous system (CNS) would need to occur. Screening for TB involves a thorough evaluation of the patient and a diagnostic test such as tuberculin skin test or interferon-gamma release assay (IGRA) [3, 4]. Screening for TB in PLWH poses extra challenges due to increased rates of false-negative tests and increased rates of extrapulmonary TB among PLWH compared to people without HIV and in those with more advanced immune suppression among PLWH [5–7].

A thorough discussion of screening and/or early diagnosis of TB is out of the scope of this review but may include symptom screening, chest radiography, C-reactive protein, and/or rapid diagnostic tests such as Xpert MTB/Rif (Xpert, Cepheid, Sunnyvale, CA, USA) in sputum or lipoarabinomannan (LAM) testing of the urine (Determine TB LAM antigen (AlereLAM), Abbott, Chicago, USA) [3, 8–10]. Ultimately, our limited understanding of the pathophysiology of TBM and inadequately sensitive diagnostic tests mean that true prevention of TBM is not a realistic systemic strategy. Thus, our efforts currently are best focused on improving delays in the presentation and diagnosis.

## Diagnosis of TB Meningitis

**Fig. 1:**
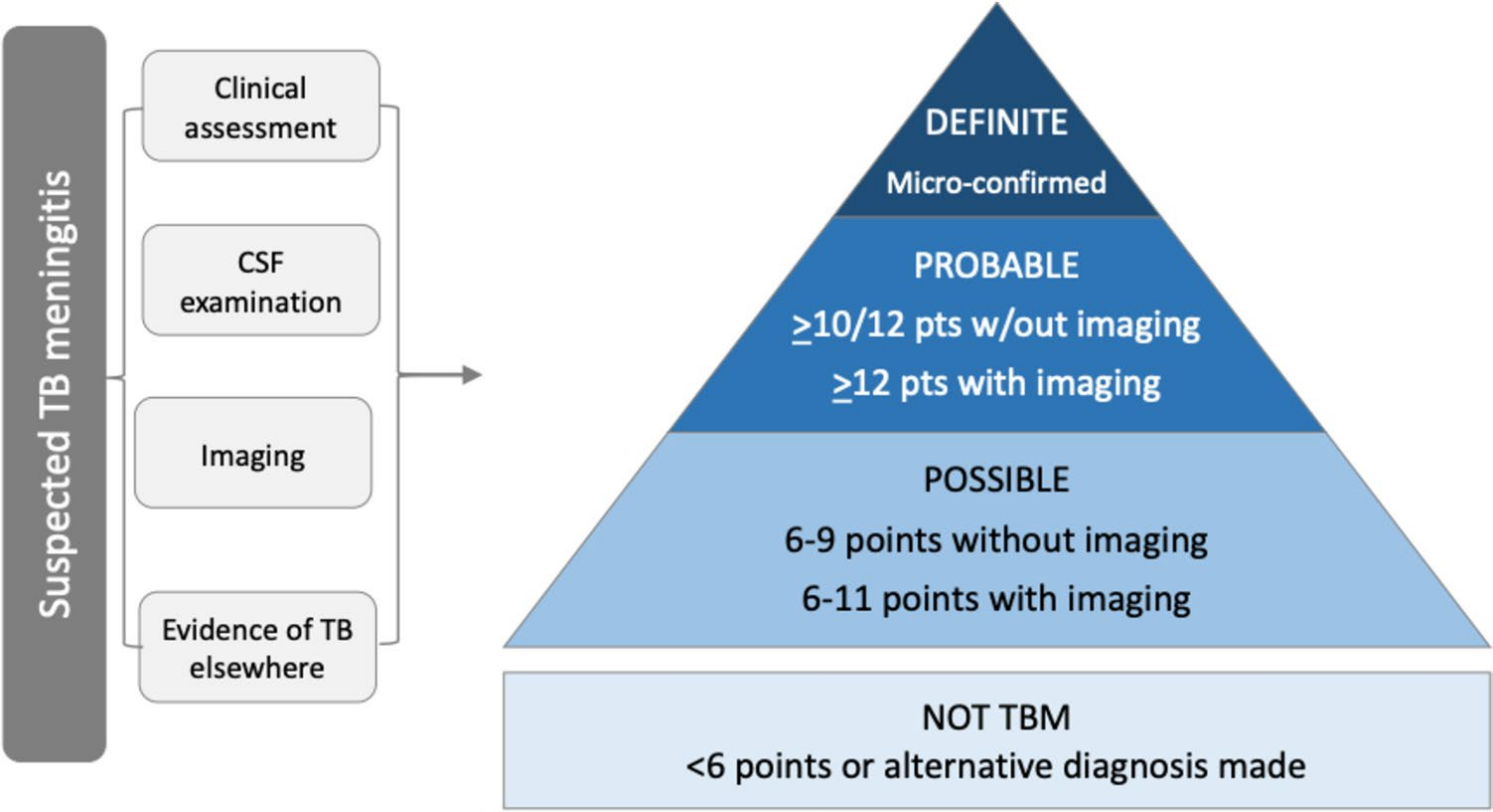
TBM uniform case definition

A uniform case definition for TBM diagnosis was created in 2010 to allow comparison of disease states between research studies (Fig. 1) [11]. The uniform case definition classifies patients as “definite,” “probable,” “possible,” and “not TBM” based on a composite score of clinical findings, CSF findings, neuroimaging, evidence of TB outside the brain, and exclusion of alternative diagnosis [11]. However, the uniform case definition lacks specificity and was designed for research, not patient care. Thus, the criteria are meant to be quite broad (depending on the inclusivity of the categories used) and may lead to inappropriate use of potentially toxic TBM therapy and missed alternative diagnoses. At the same time, delayed or missed diagnoses of TBM occur due to the inadequate sensitivity of available diagnostic tests [11, 12]. Table 1 outlines the performance and limitations of commonly used or promising diagnostic tests as well as some that are often discussed but not routinely used.

**Table 1:** Performance of commonly used, promising, or often discussed but not routinely used tests for TB meningitis in adults

CSF test	Sensitivity^*^	Specificity^*^	Time to results	Limitations	References
*Frequently used diagnostic tests*					
AFB smear	8–34%	100%^a^	Hours	Poor sensitivity in most settings	[16, 120]
Culture	32–61%	100%	2–6 weeks	Slow, lab infrastructure, costly, inadequate NPV	[22, 120, 121]
ALERE LAM	22–33%	94–96%	Minutes	Sensitivity, intra-operative variability, inadequate NPV	[31, 32]
Traditional NAAT	24–87	98–100%	Hours/days	Cost, lab infrastructure, many “in-house” tests, variable study design and targets, stringent operational conditions, technical expertise	[122]
Xpert	40–85%	97–100%	Hours	Cost, inadequate NPV, variable study design and performance	[22, 23, 51, 121, 123, 124]
Xpert Ultra	47–95%	100%	Hours	Cost, inadequate NPV variable study design and performance	[22, 23, 51, 121]
TrueNat	78–86%	100%	< 1 hour	Cost, requires technical expertise, results require confirmation	[27]
*Commonly discussed tests not commonly used*					
Adenosine deaminase	79–91%	86–91%	Days	Cost, lab infrastructure, false positives, study heterogeneity, variable test performance	[36, 125, 126]
IGRA	77–79%	91–95%	Days	Cost, lab infrastructure, false positives, indeterminate results, varied study designs and cut-points	[35, 127]
*Promising tests not yet available*					
Fujifilm SILVAMP TB LAM	52–74%	98%	1 hour	Cost, intra-operative variability, inadequate NPV	[34••]

*All sensitivity and specificity values are approximate, based on current literature with the understanding that variability occurs between studies and with local disease prevalence. ^a^Though these studies report 100% specificity, clearly there is potential for other mycobacteria to falsely cause positive results. *AFB* acid-fast bacilli, *IGRA* interferon-gamma release assay, *LAM* lipoarabinomannan, *NAAT* nucleic acid amplification test

### CSF Analysis

Persons with TBM generally have CSF lymphocyte-predominant pleocytosis (CSF white blood cell count: 10–1000 cells/μL), increased CSF protein > 45 mg/dL, and CSF:blood glucose ratio < 0.5 or CSF glucose < 2.2 mmol/L, and/or high CSF lactate [13, 14]. Of these, glucose measurement best predicts microbiologically confirmed TBM in some studies [15]. Importantly, none of these tests are specific for TBM and cases frequently occur with test values outside of the ranges above [14].

### Microbiological Tests

Visualization of CSF acid-fast bacilli (AFB) by smear microscopy is the cheapest and most widely used rapid diagnostic test for TB; however, microscopy has poor sensitivity in most settings (8%, 95% CI, 3–21% in one systematic review and meta-analysis) and as such is not reliable for the diagnosis of TBM [16]. In many settings, this is the only test available, but if more sensitive tests are available, they are preferred.

Mycobacterial culture has traditionally been considered the gold standard for the diagnosis of TBM, but turnaround time is too slow for clinical action in most cases (2–8 weeks depending on media type) [12, 17]. Yet, sensitivity is still much greater than AFB smear, 50–60%, allowing for confirmation of putative diagnoses in some cases [12]. Further, *M. tuberculosis* (*M.tb*) culture is important for phenotypic drug susceptibility testing (DST), as well as epidemiologic and sequencing-based studies.

### Molecular Tests

Nucleic acid amplification tests have significantly changed the landscape of TBM diagnosis, where available. While many centers have developed “in-house” polymerase chain reaction (PCR) tests, commercially available PCR tests exist although cost and laboratory expertise often limits implementation [14, 18]. Similarly, while loop-mediated isothermal amplification (LAMP) is appealing as it is isothermal, does not require electricity, and is highly sensitive (88–96%) against culture; LAMP requires primer design which is often limiting in most centers without appropriate laboratory expertise in test design (e.g., it is not ready to use “off the shelf” as is Xpert) [14, 18–20].

The greatest improvement has been the development of Xpert and subsequently GeneXpert MTB/Rif Ultra (Xpert Ultra). Both Xpert and Xpert Ultra are cartridge-based molecular tests with run-times < 2 h and sensitivities equivalent to (Xpert) or greater than (Xpert Ultra) *Mtb* culture [14]. Xpert was initially developed using only the *rpoB* gene to detect TB itself and gene mutations related to rifampin resistance. Xpert Ultra was developed using additional DNA probes (IS1081 and IS6110) and a larger PCR reaction chamber to improve its lower limit of detection (16 CFU/mL for Xpert Ultra versus 113 CFU/mL for Xpert) [21]. Xpert Ultra was initially studied in Uganda using samples of cryopreserved CSF and showed 95% sensitivity against definite TBM and 70% against probable or definite TBM [22]. These findings have largely been confirmed. In a 2021 Cochrane systematic review, the pooled sensitivity and specificity of Xpert Ultra against culture were 89.4% (95% Cl, 79.1–95.6%) and 91.2% (95% Cl, 83.2 to 95.7). The Xpert pooled sensitivity and specificity against culture were 71.1% (95% Cl, 62.8 to 79.1) and 96.9% (95% Cl, 95.4 to 98.0) [23]. Notably, performance can be improved through centrifugation of CSF [24]. In our opinion, the specificity values in these studies are likely to be artificially low as they counted *M.tb* DNA from CSF in the absence of culture as “false positives,” whereas such results are much more likely to reflect true TBM infection. This effect is more pronounced for Xpert Ultra given its lower limit of detection. Importantly, neither Xpert nor Xpert Ultra has adequate negative predictive value to “rule-out” TBM [25, 26].

Truenat MTB Plus (Molbio, Verna, India) is a near-care molecular test that provides rapid and accurate results. Among 76 persons with definite TBM, 32 with probable TBM and 40 non-TBM controls, Truenat MTB Plus found sensitivity of 78.7% compared to 67.6% with Xpert Ultra. If only definite TBM cases were compared, Xpert Ultra had 96% sensitivity versus 85.5% for Truenat MTB plus [27]. Both modalities detected cases; the other missed. While promising, there is only one published study of this technology for TBM to date [27]. Additionally, Truenat MTB Plus requires technical expertise for sample preparation and DNA extraction although the assay is stable at high temperatures and is battery-operated, ideal for settings where the electrical supply may be unreliable.

Metagenomic next-generation sequencing (mNGS) has the potential to detect not only *M.tb*, but also other potential pathogens that may mimic TBM in an unbiased fashion. One study showed sensitivity of 50.8% versus definite or probable TBM [28•]. Interestingly, if probable cases with alternative pathogens detected by mNGS were reclassified as not TBM, sensitivity increased only to 54.2%, although in those three cases, finding that alternative pathogen would have been significant [28•].

The use of CRISPR-mediated detection of circulating *M.tb* cell-free DNA (CRISPR-MTB) provides additional potential for diagnosis. One 2019 study of this technology used on CSF found 73% sensitivity among 26 cases deemed TB meningitis by a non-standard clinical definition versus 54% for Xpert and 23% for culture [29]. Whether these results can be replicated in other studies and populations using standard definitions needs to be determined.

### Lipoarabinomannan (LAM) Antigen Tests

The AlereLAM test is commonly used on urine to diagnose TB in people with advanced HIV [30]. This test is attractive as a diagnostic test for TBM as it is a rapid lateral flow test that does not require significant expertise or a stable electrical supply. Yet, AlereLAM performance on CSF to diagnose TBM is poor. In 2019, an antemortem prospective cohort study in Uganda found a sensitivity of 24–33% (varied by reference standard); other centers have obtained similar results [31, 32].

The Fujifilm SILVAMP TB-LAM test (FujiLAM) is a novel urine test that has improved sensitivity in urine versus AlereLAM and uses novel LAM epitopes and silver amplification step [33]. Thus far, only one study has been performed in TBM using CSF samples [34••]. Among 34 persons with definite TBM, 24 with probable TBM, and 43 controls, sensitivity was 52% compared to 55% for Xpert Ultra [34••]. Both tests detected cases the other test did not; however, importantly, it is not clear if cases detected only by FujiLAM were true positives or false positives due to cross reactivity.

### Host-Derived Rests—Biomarkers, RNA Transcripts, and Antibody Tests

There has been interest in IGRA on CSF to diagnose TBM. A recent systematic review and meta-analysis found that among eight studies including 694 samples, sensitivity was 77% (95% CI, 56–90%) and specificity 91% (95% CI, 85–95%) [35]. Sensitivity is clearly variable by study, and specificity remains too low such that results are unreliable. Further, use is limited by relatively high volumes of CSF required (4 mL), laboratory infrastructure, cost, and frequency of indeterminate results.

Adenosine deaminase is another biomarker that has been studied on CSF for TBM with a recent meta-analysis of 43 studies and over 5000 patients finding pooled sensitivity of 86% (95% CI, 86–91%) and specificity 89% (95% CI, 86–91%) [36]. Limitations such as cost, laboratory infrastructure, and inadequate specificity (particularly in PLWH) have limited its use, and significant heterogeneity in study design has made interpretation of study results difficult, despite significant numbers having been studied [14, 37].

There are clear differences in RNA transcripts seen between CSF samples from those with TBM and healthy controls. Importantly, RNA sequencing has also shown differences between CSF collected from lumbar and ventricular CSF which would have implications were these transcript patterns to be used in the diagnosis of TBM. Whether this can lead to a diagnostic tool is so far unclear, but there has been promise in this area where a machine learning classifier was used to harness RNA transcript data to explore this possibility [28•, 38].

CSF cytokines and chemokines have been of interest, particularly interferon gamma where one meta-analysis and systematic review found sensitivity of 86% (95% CI, 76–92%) and specificity of 92% (95% CI, 82–96%) among 225 samples and four included studies [35]. Numerous CSF cytokines and chemokines have been found to be higher or lower in aggregate in TBM than comparators, but in general, this is an area where further research is needed to understand whether they might have a role as part of a diagnostic test schema [12, 39]. Additionally, while tryptophan metabolism processes and markers are of keen interest in TBM as prognostic markers and potential therapeutic targets, their potential as diagnostic tools is unclear [40]. Other immune biomarkers such as delta-like 1 ligand, fetuin, and vitamin D binding protein have been evaluated for TBM but performance was poor [41]. Various antibodies such as anti-M37Ra, anti-antigen 5, or anti-M37Rv have also been evaluated, but despite high sensitivities in some studies, these are not used due to heterogeneity in performance and study design across studies and a lack of commercial assays [42].

### Combination of Modalities

Ultimately, it may be that no single test gives adequate diagnostic accuracy for TBM. Combination approaches using either multiple different bacilli-focused targets (for example molecular plus LAM) or molecular/microbiological approaches added to host-based approaches may have the potential to improve performance [12, 14, 32]. For instance, Siddiqi and colleagues combined Xpert, CSF AlereLAM, CSF glucose, and CSF protein for an area under the receiver operator curve of 0.90 [32]. Ramachandran and colleagues combined their machine learning classifier from RNA sequencing data with mNGS data for 89% (8/9) sensitivity and 87% (65/75) specificity. These studies are hypothesis driving; each approach needs further study and new tests need to be considered, but the ultimate best diagnostic scenario may be a combination approach.

### Imaging

Traditional imaging modalities like chest radiography and abdominal sonography are used to look for evidence of TB outside the brain as highlighted in the uniform case definition [11]. Neuroimaging may provide additional information although availability remains a major barrier and many settings where TB meningitis is common, particularly for magnetic resonance imaging (MRI).

Brain computer tomography (CT) may show infarctions, hydrocephalus, tuberculomas, and/or basal exudates. In combination, these are suggestive of TBM although children commonly also have normal CT studies, and none of these findings were present in > 40% of initial CT imaging among 209 cases of CNS TB [43, 44]. Similarly, among 452 initial brain MRI examinations in persons with CNS TB, basal meningeal enhancement, hydrocephalus, tuberculoma, and infarction were each commonly seen, but none of these in more than 41% [44].

### TBM Testing by HIV Serostatus

Diagnosis of TBM can be more challenging in PLWH, especially for those with advanced HIV. Several standard tests for TB diagnosis, including tuberculin skin tests and interferon-gamma release assays, as well as basic CSF analysis and culture, may perform worse in PLWH [6, 45, 46]. For instance, while lymphocytic pleocytosis is typical (and seen regardless of HIV status in some studies), Thwaites and colleagues found lower CSF WBC among PLWH compared to people without HIV (median 152 × 10^3^ cells/L vs 356 × 10^3^ cells/mL [47]. Similarly, CSF protein may be lower in PLWH than in people without HIV [48]. The theme in considering basic CSF studies is that, in general, “classic” CSF findings are less frequent in those with HIV than those without. For instance, only 64% of PLWH with culture-confirmed TBM had typical CSF findings in one study [49] and basic CSF studies within normal ranges occur [45]. Yet, other adjunctive tests such as urine AlereLAM for TB diagnosis are most helpful in advanced HIV (50). Further, in one study, whether or not basic CSF testing, AFB smear, in-house PCR, or Xpert in CSF performed better in those with or without HIV was unclear although culture did perform better in those without HIV [46]. Another study found better performance of Xpert and Xpert Ultra in PLWH than people without HIV on CSF for TBM diagnosis [51]. Ultimately, population, host, and microbe factors may make broad distinctions based on HIV status of unclear utility.

### Diagnosis of Pediatric TBM

Pediatric-specific diagnostic test performance data exists for some tests, but publications are, in general, less common. A 2022 study found 50% (2/4) sensitivity and 91% (130/143) specificity in 149 children tested with Xpert Ultra and 18% (3/17) sensitivity with 99% (174/175) specificity among 192 children tested with Xpert using definite, probable or possible TBM as a composite reference standard [52]. Importantly, definite TBM in this study was only culture-positive, and cases positive only by Xpert or Xpert Ultra were considered falsely positive, and so its possible that specificity is artificially low, particularly for Xpert Ultra. This is the only study of Xpert Ultra in children published to date. There have also been studies investigating CSF immune biomarkers in children. One such study found an AUC of 0.89 with a combination of vascular endothelial growth factor-A, IFNg, and myeloperoxidase [53]. Further validation in pediatric populations is needed, and it seems unlikely these findings could be applied to adults unless validated in that population as well given differences in the adult and pediatric immune responses.

## Treatment of Tuberculosis Meningitis

### Current First-Line and Alternative Treatment Options

Early initiation of anti-tuberculosis treatment in patients for whom there is clinical suspicion of TBM is essential as a definitive microbiological diagnosis remains difficult and delayed treatment is strongly associated with poor outcomes [54]. No data support altering the choice or duration of anti-tuberculosis therapy for PLWH, although special considerations must be made in light of drug-drug interactions between antiretrovirals and TB drugs [55–57].

The optimal drug regimen and duration of treatment for TBM are not well defined as chemotherapy is based largely on expert opinion, observational studies, and extrapolation of data for the treatment of pulmonary TB [55, 58, 59]. The earliest drug used for the treatment of TBM, streptomycin, was associated with poor survival and high resistance rates—improved to some degree with the addition of para-amino salicylic acid [58, 60]. Patient outcomes further improved with the introduction of isoniazid, a drug with potent bactericidal activity, a more favorable toxicity profile and excellent CNS penetration [58, 61]. Subsequently, rifampin and pyrazinamide—both with sterilizing activity—were added [55, 58, 61–64].

The WHO recommends a combination of isoniazid, rifampin, pyrazinamide, and ethambutol as first-line treatment of TBM during the 2-month intensive phase of therapy, followed by isoniazid and rifampin for an additional 7 to 10 months [65•]. In some settings, streptomycin is used in place of ethambutol, though both demonstrate limited CSF penetration, and streptomycin is poorly tolerated, contraindicated in pregnancy, and has a high potential for resistance [59, 61]. Some centers advocate for ethionamide instead of ethambutol owing to its favorable safety profile and with good CSF penetration in both healthy and inflamed meninges [59].

Patients with multi-drug-resistant TBM, defined as resistance to at least isoniazid and rifampin, are at high risk for treatment failure and death [59, 62]. The choice of second-line drugs is informed by probable drug susceptibility and CSF penetration, and so moxifloxacin, levofloxacin, and linezolid are attractive options [59, 66]. Further description of potential alternative strategies, including agents active in drug-resistant TBM are discussed below.

### Improving TBM Antibacterial Treatment

There are two broad therapeutic strategies to improve outcomes in TBM: enhanced bacterial killing through intensified antibiotic therapy and more targeted host-directed approaches to reduce inflammation. Enhanced bacterial killing may be achieved by optimizing the use of existing drugs (e.g., at higher doses), repurposing agents with activity against *M.tb*, and using new drugs and treatment combinations. Regardless of the therapeutic strategy, the central consideration for the selection of TBM regimens is drug potency, which requires both in vitro bactericidal and sterilizing activity and attainment of efficacious exposures at the site of infection. Complex factors influence drug concentrations at the site of disease in TBM, including properties of the drug (e.g., lipophilicity), extent of plasma and CSF protein binding, influence of blood-brain barrier transporters, blood-brain barrier integrity, and dynamic inflammation of CNS structures. Besides potency, the introduction of new regimens in TBM will depend on clinical efficacy and safety profiles, plus propensity for pharmacokinetic drug-drug interactions. These characteristics are known for several existing and new anti-tuberculosis drugs and can be used to prioritize agents for evaluation in novel TBM regimens (Table 2).

**Table 2:**
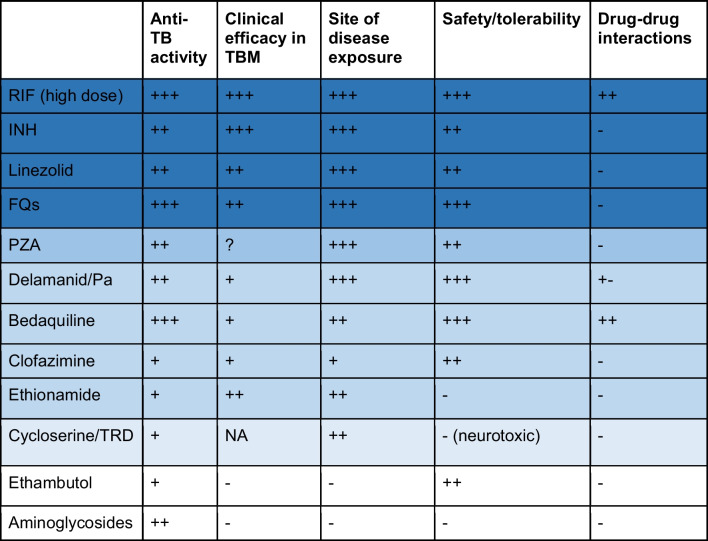
Characteristics of registered anti-tuberculosis drugs for use in TBM

Darker shade, more favorable characteristics overall. Ranking of drugs for each category. Anti-tuberculosis activity: +++, potent bactericidal sterilizing activity in vitro and in vivo; ++, moderate mainly related to EBA; +, weak activity at tolerable doses. Clinical efficacy in TBM: +++, benefit in randomized controlled trials; ++, benefit in non-randomized studies; +, benefit in case reports; -, no clinical benefit demonstrated; NA, not assessed. Site of disease exposure: +++, potentially therapeutic concentrations in brain parenchyma from animal models and/or non-invasive human studies; ++, detectable concentrations in brain parenchyma or CSF but possibly below therapeutic thresholds or only at toxic doses; +, detectable at very low concentrations; -, undetectable. Safety/tolerability: +++, well tolerated at optimized doses with low toxicity potential; ++, generally well tolerated but may have treatment-limiting AEs; -, poorly tolerated and frequent treatment-limiting AEs. DDIs: +++, high potential, treatment limiting, perpetrator; +, victim of DDIs; -, no clinically relevant DDIs, DDI: drug-drug interactions

Although effective regimens for pulmonary TB may not translate into clinical effectiveness in TBM, one way to approach regimen design is to apply principles for drug selection described above to promising combinations being evaluated for treatment shortening in pulmonary TB. Three broad strategies are currently being pursued for pulmonary TB: optimized dose rifamycin-based regimens, bedaquiline-based regimens, and fluoroquinolone-containing regimens.

All current rifamycin-based treatment shortening regimens for pulmonary TB contain both isoniazid and pyrazinamide, with the addition of either linezolid, a nitroimidazole, or clofazimine. Several trials of high-dose rifampin-isoniazid regimens are either completed or underway for TBM [67–70]. Isoniazid may have a critical role in TBM given the observation that isoniazid exposures were associated with survival and that the addition of a fluoroquinolone was protective among patients with isoniazid monoresistance in a large clinical trial [71–73]. Optimized pyrazinamide dosing may also enhance bacterial killing because of synergy with rifampin and isoniazid, action on extracellular bacteria (including in caseum), and excellent CNS exposure in animal models [74]. The nitroimidazoles—delamanid and pretomanid—have potent early bactericidal activity and may also achieve therapeutic concentrations in the CNS, making them attractive agents for evaluation in TBM regimens [75–78]. Clofazimine is a cationic amphiphilic (possesses both hydrophilic and hydrophobic elements) drug with extensive distribution and tissue accumulation and is likely to equilibrate rapidly into lipid-rich brain tissue [79]. Clofazimine has been measured at low concentrations in the CNS in a murine experiment but was undetectable in CSF from five patients with TBM [80–83•].

Can rifamycin-free regimens be contemplated for TBM? Bedaquiline-based regimens being evaluated for pulmonary TB contain companion drugs (including isoniazid, pyrazinamide, nitroimidazoles, and linezolid) with excellent anti-TB activity and may have favorable pharmacokinetics characteristics for use in TBM. Other observations suggest that rifampin may not be an ideal drug for TBM. First, rifampin may not achieve adequate site of disease concentrations at current doses, and although higher doses lead to higher CSF concentrations, this has not yet conclusively led to survival benefit, though clinical trials are ongoing. Second, drug-drug interactions with rifamycins are problematic and often treatment-limiting, including with bedaquiline and novel diarylquinolines. Third, rifampin-resistant TBM is under-recognized and severe, and a rifamycin-free regimen would address this. Finally, rifamycin-free regimens perform better—in terms of cure and bactericidal activity—in mouse models for pulmonary TB, a tantalizing prospect for TBM where early bacterial killing is thought to be critical. The core drug in these regimens, bedaquiline, has pharmacological characteristics suggesting a high likelihood of distribution into the CNS, and despite high protein binding, it has been detected in CSF from patients with TB [84, 85].

###  Corticosteroids in TBM

The mortality and morbidity observed in TBM are due to an inflammatory process set off by the *M.tb* in the CNS and resultant dysregulated host immune response [86]. Corticosteroids have a significant role in immune modulation [87].

The breakdown of mycobacteria in the subarachnoid space triggers the production of inflammatory cytokines, blood-brain barrier disruption, leaking of proteins, and buildup of inflammatory exudate. The injury that results from this pathological cascade leads to common complications of TBM such as vasculitis-induced infarcts and obstructive hydrocephalus. Corticosteroids reduce inflammation by inhibiting the synthesis of inflammatory cytokines and stabilizing the blood-brain barrier [88]. Adjunctive corticosteroids have been shown to reduce death by ~40% but not disability in TBM overall [88, 89]. The survival benefit of glucocorticoids in HIV-TBM co-infection, however, is less certain, and results from a randomized controlled trial investigating this question were recently published and did not show a benefit from corticosteroids [90••].

### Other Host-Directed Therapies

Cerebral infarctions are common in TBM and associated with higher mortality [91, 92]. Aspirin, a widely available and inexpensive drug, prevents stroke at low doses (75–150 mg/day) through inhibition of thromboxane-A2 and prevention of platelet aggregation [93]. At high doses (> 600 mg/day), aspirin exhibits anti-inflammatory properties through inhibition of pro-inflammatory cytokines including TNF-alpha [94]. Although there have been studies evaluating aspirin at doses ranging from 75 to 1000 mg with variable designs and sizes, it remains unclear whether there is a benefit from aspirin in TBM and further studies are ongoing [95–99].

Other host-directed immunomodulatory therapies, including thalidomide, TNF-alpha inhibitors, and interleukin-1 receptor antagonists, are used with increasing frequency in select patients with TBM who demonstrate an immune-mediated paradoxical response that is refractory to corticosteroids or to tapering of corticosteroids [95, 100–103]. Most of these cases are described in individuals without HIV co-infection, although a similar host-directed approach has been used in PLWH with TBM immune reconstitution inflammatory syndrome (IRIS) [104, 105].

### Antiretroviral Therapy in TBM

There is overwhelming evidence that ART initiation has reduced mortality in PLWH and TB, in studies that include persons with TBM [106]. Yet, among 253 Vietnamese adults with HIV and TBM, there were more grade 4 adverse events among HIV/TBM patients who were initiated on ART within 1 week of antitubercular treatment compared to patients whose initiation ART was deferred to 8 weeks [107]. Second, delaying ART initiation among a South African HIV cohort with microbiologically confirmed TBM was associated with reduced TBM-IRIS [108]. The results of these studies informed the WHO recommendation to defer ART initiation to 8 weeks after TBM treatment initiation among patients with HIV/TBM co-infection [109].

Unfortunately, other CNS infections are great mimickers of TBM, so in the absence of microbiological confirmation, it is difficult to distinguish TBM from other non-cryptococcal (ART is also delayed in cryptococcal meningitis) causes of meningitis. Ramachandran and colleagues found 17 different pathogens in CSF of HIV patients who had previously been classified as suspected [28•]. This finding poses a challenge to the current clinical practice where lifesaving ART is withheld from PLWH with suspected TBM, with no confirmation of mycobacterial infection in the CNS, and severe immunosuppression until ART is initiated at week 8. The risk of progression of an alternative opportunistic infection needs to be considered in the context of delaying ART against the risk of TBM-IRIS—both results can portend poor outcomes. Ultimately, this conundrum highlights the need for rapid, accurate diagnostic tests for TBM in PLWH.

### Drug-Drug Interactions

When ART is used in those with TBM, a number of drug-drug interactions must be considered. Rifampin is a strong cytochrome P450 (CYP3A4) inducer; hence, caution must be taken regarding the choice of ART regimen administered [57]. Doubling the dose of dolutegravir is currently recommended when co-administered with rifampin, although recent data found virological outcomes at 6 months to be equal in those given single dose or double dose dolutegravir during TB treatment, and so dose doubling may not be necessary [110, 111]. Co-administration of rifampin with protease inhibitors is not advised given the significant drop in serum concentrations of the protease inhibitor even with a ritonavir boosting regimen hence the potential for low therapeutic effect and development of resistance [57]. Double dosing of the protease inhibitor has been used where no other options exist but is poorly tolerated. Concomitant treatment of rifampin induces metabolism of nevirapine but less so for efavirenz [112].

### Treatment of Pediatric TBM

The current WHO recommendation is for isoniazid, pyrazinamide, rifampin, and ethambutol for 12 months. However, informed by a systematic review, WHO recently listed 6 (HIV negative) or 9 (PLWH) months total with higher doses of isoniazid, rifampin, and pyrazinamide and substituting ethionamide for ethambutol as alternative durations [113, 114]. As in adults, there are recent and ongoing trials hoping to find more effective and less toxic treatment regimens. TBM-KIDS, an open-label, phase 2, randomized clinical trial which enrolled 37 children in India and Malawi was recently published [115]. All participants received isoniazid and pyrazinamide and one of three regimens: 1) high-dose rifampicin (30 mg/kg) and ethambutol, 2) high-dose rifampicin plus levofloxacin, or 3) standard-dose rifampicin plus ethambutol. There were trends towards more adverse events but better neurological outcomes in the high-dose rifampicin arms, but a larger trial is needed.

### Therapeutics—Areas of Uncertainty and Ongoing Trials

A series of phase II trials in Indonesia evaluated the pharmacokinetics:pharmacodynamics (PK:PD) and safety of higher doses of rifampicin administered intravenously and orally [116, 117]. In Uganda, a phase II open-label randomized controlled trial (ISRCTN42218549) assessed the safety and pharmacokinetics of high-dose rifampin (given orally at 35 mg/kg/day or intravenously at 20 mg/kg/day) in comparison to the standard of care anti-TB medications in PLWH. In the standard of care arm, around a third had undetectable rifampin concentrations. The per oral arm taking rifampin at 35 mg/kg/day achieved the high CSF total exposure, with all participants having rifampin above the minimum inhibitory concentration [68•]. This 35 mg/kg dose is now being investigated in a phase 3 multi-site study (HARVEST, ISRCTN15668391) [67].

In a retrospective cohort study, Feng et al. demonstrated that TBM patients who had baseline Medical Research Council (MRC) grade 2/3 and received adjunctive linezolid (a drug initially only used in treatment of multi-drug-resistant TB) achieved improvement in their consciousness faster within the first 4 weeks, quicker fever resolution, faster improvement in the CSF:serum glucose ratio, and faster reduction in CSF inflammation [118]. More studies have now been designed to evaluate the pharmacokinetics and pharmacodynamics of adjunctive linezolid. Adjunctive linezolid for treatment of TBM (ALTER study) is a phase II randomized open-label trial having three intervention arms through the first 4 weeks: arm 1 receiving adjunctive linezolid (1200 mg/day) plus high-dose rifampin (35 mg/kg/day); arm 2 receiving adjunctive linezolid (1200 mg/day) plus standard of care TB medications; arm 3 receiving only high-dose rifampin (35 mg/kg/day), with all these groups receiving standard dosing of isoniazid, pyrazinamide, and ethambutol. The comparative arm for this study is being the standard of care [69]. The primary goals of the study are to assess tolerability and pharmacokinetics of linezolid, the association with grade >3 adverse events at 4 weeks, and to look at functional status at 4, 12, and 24 weeks (NCT04021121).

The ongoing intensified trial (INTENSE) trial is a phase 3 randomized controlled trial assessing both the role of adjunctive linezolid, high-dose rifampin and aspirin 200 mg in the first 8 weeks for treatment of TBM (NCT04145258). The ongoing short intensive treatment for children with TBM (SURE) trial is a randomized controlled trial investigating the use of higher doses of rifampin (30 mg/kg vs 15 mg/kg), isoniazid (20 mg/kg), and pyrazinamide (40 mg/kg) in combination with levofloxacin (20 mg/kg) [119]. This trial also included a second randomization versus placebo for aspirin. Table 3 summarizes major ongoing and recently completed studies.

**Table 3:** Major ongoing and recently completed TBM clinical trials

Name	Phase/design	*N*	Countries	Antimicrobial interventions	Host-directed interventions*	Start–end
Ongoing						
INTENSE NCT04145258	III Factorial	768 (192 / arm)	Cote d’Ivoire Madagascar Uganda South Africa	1. R 35 mg/kg for 8 weeks + LZD 1200 mg for 4 wks then 600 mg for 4 wks + standard H, Z, E dosing for 8 wks, standard continuation phase. 2. WHO standard regimen	1. Aspirin 100 mg every other day for 8 wks 2. Placebo for 8 wks	2020–2023
HARVEST ISRCTN15668391	III Parallel	500	Uganda South Africa Indonesia	1. R 35 mg/kg for 8 weeks, standard H, Z, E for 8 wks, standard continuation phase 2. WHO standard regimen	Nil	2021–2024
ALTER NCT04021121	II Factorial	60 (15 / arm)	Uganda	1. R 10 mg/kg 2. R 10 mg/kg + LZD 1200 mg 4 wks 3. R 35 mg/kg 4. R 35 mg/kg + LZD 1200 mg 4 wks All with standard continuation phase	Nil	2021–2023
SIMPLE NCT03537495	II Parallel	36	Indonesia	1. R 35 mg/kg 2. R 35 mg/kg + LZD 600 mg 2 wks 3. R 35 mg/kg + LZD 1200 mg 2 wks All with standard continuation phase	Nil	2021–2023
LAST ACT NCT03100786	III Parallel	640	Vietnam HIV-negative only	Nil	1. LTA4H TT-genotype: dexamethasone 2. CC or CT genotype: placebo or dexamethasone	2018–2022
SURE ISRCTN4089906	III Factorial	400	Vietnam Uganda India Zambia Zimbabwe Children, 29 days–15 years	1.WHO standard regimen 2. R 30 mg/kg + H 20 mg/kg +Z 40 mg/kg + Lfx 20 mg/kg for 6 months	1. Aspirin 20 mg/kg for 8 wks 2. Placebo for 8 wks	
Completed						
ACT HIV NCT03092817	III Parallel	520	Vietnam Indonesia HIV-positive only	Nil	1.Dexamethasone 2. Placebo	2017–2022
LASER-TBM NCT03927313	IIb parallel	100	South Africa	1. R 10 mg/kg 2. R 35 mg/kg + LZD 1200 mg 4 wks then 600 mg 4 wks	1. Aspirin 1000 mg 6 wks added to half of the intensified arm	2019–2021
RifT ISRCTN42218549	II	60	Uganda	1.R 10 mg/kg 2. R 35 mg/kg 3. R 20 mg/kg intravenous	Nil	2019–2020
ReDEFINe NCT02169882	II	60	Indonesia	1.R 450 mg 2. R 900 mg 3. R 1350 mg	Nil	2014–2017
TBM-KIDS NCT02958709	II	37	India Malawi Children 6 months–12 years	1: R 30 mg/kg + E 2: R 30 mg/kg + Lfx 3: Standard WHO regimen	Nil	2017–2019

*R* rifampin, *H* isoniazid, *Z* pyrazinamide, *E* ethambutol, *LZD* linezolid, *Lfx* levofloxacin, *wks* weeks

*Unless otherwise indicated corticosteroids are given to all as recommended by WHO and control regimens are WHO-recommended R 10 mg/kg, H 5 mg/kg, Z 30 mg/kg, E 20 mg/kg 2 months, followed by RH for 7–10 months

## Summary

TBM remains a deadly disease, but one where real progress has been made in recent years. Diagnosis has improved dramatically in locations where rapid molecular assays like Xpert Ultra are available. Yet, affordability and infrastructure requirements limit implementation. Thus, in many lower-income settings, AFB smear is the only test available and so many cases go undiagnosed. A number of promising tests are being considered, including FujiLAM as an additive test. For these tests to meet their promise, affordability and accessibility will be key.

There are also a number of promising areas related to treatment that have the potential to improve outcomes in the near future. Host-directed therapies clearly have a major role in TBM in general, but to truly harness their power, we need to better understand which populations benefit (or not), and to consider alternatives (or additions) to corticosteroids. In the coming years, we will gain a better understanding of what, if any, roles high-dose rifampin, delamanid, pretomanid, bedaquiline, linezolid, fluoroquinolones, clofazimine, and other agents might have as part of combination regimens for TBM. The good news is that many of these agents are now actively being studied.

There is sufficient momentum and research effort currently that one can reasonably hope that TBM might be rapidly and accurately diagnosed in the next decade in most settings, and that the regimens started after a prompt diagnosis will be more tailored to CNS tuberculosis, with better outcomes. For that to occur, current efforts will need to be maintained or improved, but the progress thus far is clear.
